# Supplementary material for: Safety of Chuna Manipulation Therapy in 289,953 Patients with Musculoskeletal Disorders: A Retrospective Study
Source: Healthcare (Basel). 2022 Feb 2;10(2):294. doi: 10.3390/healthcare10020294 (PMC8871927; doi:10.3390/healthcare10020294)
Supplement: Supplementary file 1 [file healthcare-10-00294-s001.zip › healthcare-1550262-supplementary.pdf]

Supplementary Material

Table S1. Cohen's kappa coefficients for concordance between the actual observed and chance agreements in different hospitals.

Hospital	Actual observed agreement	Chance agreement	Kappa (95% confidence interval)
Gangnam	0.995	0.957	0.877 (0.739–1.000)
Bucheon	1	0.990	1.000 (1.000–1.000)
Daejeon	1	0.994	1.000 (1.000–1.000)
Haeundae	1	0.997	1.000 (1.000–1.000)
Ulsan	1	0.965	1.000 (1.000–1.000)
Gwanghwamoon	0.992	0.863	0.943 (0.865–1.000)
Ansan	1	0.920	1.000 (1.000–1.000)
Nowon	0.996	0.960	1.000 (1.000–1.000)
Gwangju	1	0.974	0.907 (0.779–1.000)
Daegu	Perfect agreement		1.000 (1.000–1.000)
Jamsil	Perfect agreement		1.000 (1.000–1.000)
Cheongju	Perfect agreement		1.000 (1.000–1.000)
Suwon	Perfect agreement		1.000 (1.000–1.000)
Incheon	Perfect agreement		1.000 (1.000–1.000)

Table S2. Causality scale for Chuna treatment.

Causality term	Assessment criteria (WHO-UMC)	Adjusted by Chuna treatment
Certain	- Event or laboratory test abnormality, with plausible time relationship to drug intake<br>- Cannot be explained by disease or other drugs<br>- Response to withdrawal plausible (pharmacologically, pathologically)<br>- Event definitive pharmacologically or phenomenologically (i.e., an objective and specific medical disorder or a recognised pharmacological phenomenon)<br>- Rechallenge satisfactory, if necessary	- Plausible time relationship between therapeutic intervention and AE<br>- Cannot be explained by other procedures, drugs, or diseases<br>- Response to discontinuation of the therapeutic intervention plausible<br>- Symptoms recur upon resuming procedure or rechallenge (if necessary)
Probable/ Likely	- Event or laboratory test abnormality, with reasonable time relationship to drug intake<br>- Unlikely to be attributed to disease or other drugs<br>- Response to withdrawal clinically reasonable<br>- Rechallenge not required	- Reasonable time relationship between therapeutic intervention and AE<br>- Unlikely to be attributed to other drugs, procedures, or diseases<br>- Response to discontinuation of the therapeutic intervention clinically reasonable<br>- Rechallenge not required
Possible	- Event or laboratory test abnormality, with reasonable time relationship to drug intake<br>- Could also be explained by disease or other drugs<br>- Information on drug withdrawal may be lacking or unclear	- Reasonable time relationship with the therapeutic intervention<br>- Can be explained by other procedures, drugs, or diseases<br>- Symptoms of treatment discontinuation are not observed or information is lacking
Unlikely	- Event or laboratory test abnormality, with a time to drug intake that makes a relationship improbable (but not impossible)<br>- Disease or other drugs provide plausible explanations	- Improbable time relationship with the therapeutic intervention<br>- Explained by other procedures, drugs, or diseases
conditional/ Unclassified	- Event or laboratory test abnormality<br>- More data for proper assessment needed, or<br>- Additional data under examination	- Additional and more appropriate testing required.
Unassessable/ Unclassifiable	- Report suggesting an adverse reaction<br>- Cannot be judged because information is insufficient or contradictory<br>- Data cannot be supplemented or verified	-Data are inadequate or unavailable. Data are contradictory

CMT: Chuna manipulation therapy, AE: adverse events, WHO-UMC: World Health Organization-Uppsala Monitoring Centre

Table S3. Adverse events associated with increasing musculoskeletal pain.

Type of visit	Sex	Age, years	Comorbidity	Disease classification	Other treatments	CMT session(s)	AE symptoms	Imaging data before first CMT session	Imaging data after onset of AE	Severity	Causality	Post-AE treatment	Follow-up result
Outpatient	F	30	C-spine HIVD	Sprain and strain of cervical spine	CMT, acupuncture, pharmacopuncture, cupping, herbal medicine	2	Exacerbated upper limb Bi-jeung	C-spine MRI (11.06.22) taken at another hospital showed severe cervical disc herniation; referred for surgery	C-spine MRI taken at another hospital showed no changes (as recorded on the chart)	2	3	Herbal medicine (Galgeuntang), cervical brace prescribed	Bi-jeung continued 2 months later; no further follow-up data
Outpatient	M	11	-	Injury to multiple structures of knee	CMT, acupuncture, pharmacopuncture, cupping	1	Exacerbated knee joint pain	None	None	1	3	Observation of progress	No follow-up data
Outpatient	M	38	Tricuspid insufficiency, left ankle fracture; OP	Intervertebral disc disorder, unspecified	CMT, acupuncture, pharmacopuncture, cupping, herbal medicine	45	Exacerbated lower back and pain in left hip and sole of left foot	L-spine MRI; disc bulge at L5-S1	L-spine MRI; No abnormal findings	1	3	Continue current treatment and observation of progress	Mild improvement of pain until 18 months later; no further follow-up data

Outpatient	M	71	Parkinson's disease, constipation	Intervertebral disc disorder, unspecified	CMT, acupuncture, pharmacopuncture, cupping, herbal medicine	3	Exacerbated right femoral pain	Both hip AP and FROG leg radiographs; osteoarthritis in the right hip joint; R/O AVN in the right femoral head	AVN diagnosed based on MRI, taken at another hospital	3	3	Surgery	No follow-up data
Inpatient	M	41	Tympanitis	Sprain and strain of lumbar spine	CMT, acupuncture, pharmacopuncture, cupping, herbal medicine	11	New TMJ pain	None	Radiograph of mandible (18.01.24): facial tilting Radiograph of TMJ (18.01.24): No limitation on either side	2	3	Acupuncture therapy	Symptoms alleviated 1 week later
Outpatient	F	52	Hypertension	Cervicalgia due to intervertebral cervical disc disorder(M50.-), cervical region	CMT, acupuncture, cupping	1	Exacerbated right ankle pain	Whole spine radiograph: mild scoliosis in thoracic and lumbar spine	None	1	3	Observation (current treatment continued for lumbar and cervical areas; excluding CMT)	Mild improvement of ankle pain 5 weeks later; subsequent follow-up data lacking
Outpatient	F	19	-	Intervertebral disc disorder, unspecified	CMT, acupuncture, pharmacopuncture,	24	Increased lower back and lower	L-spine MRI (09.12.29): disc extrusion at L4-5	L-spine MRI (11.07.25): disc extrusion at L4-5	2	3	Existing Korean medicine treat-	Surgery 1 year later.

					cupping, herbal medicine		limb radiating pain	disc protrusion at L5-S1	disc protrusion at L5-S1			ment continued and N-block performed	
Outpatient	M	40	-	Other specified intervertebral disc disorders	CMT, acupuncture, pharmacopuncture, cupping, herbal medicine	8	Increased lower back and lower limb radiating pain	L-spine MRI (10.01.17): disc extrusion with inferior migration in L4-5, possibly compressing left L5 nerve root Disc extrusion in L5-S1	None	2	3	Existing Korean medicine treatment continued and nerve block performed	3 rounds of nerve block with herbal medicine over 3 months; pain reduced
Outpatient	M	33	-	Intervertebral disc disorder, unspecified	CMT, acupuncture, pharmacopuncture, cupping, herbal medicine	17	Increased lower back and lower limb radiating pain	L-spine MRI (10.03.18): bulging disc at L3-4; disc extrusion at L4-5	None	2	3	Existing Korean medicine treatment continued and nerve block performed	Herbal medicine added for 6 months; pain reduced
Outpatient	M	42	DM	Other specified intervertebral disc disorders	CMT, acupuncture, pharmacopuncture, cupping, herbal medicine	12	Increased lower back and lower limb radiating pain	L-spine MRI (10.04.12): disc protrusion at L5-S1; bulging disc in L3-4 and L4-5	None	2	3	Existing Korean medicine continued with oral Western analgesics	Herbal medicine added for 4 months; pain reduced

Outpatient	M	28	L-spine HIVD	Intervertebral disc disorder, unspecified	CMT, acupuncture, pharmacopuncture, cupping, herbal medicine	2	Increased lower back and lower limb radiating pain	L-spine MRI (08.11.13): L4-5 and 5-S1 disc extrusion, indentation of both L5 and S1 roots	L-spine MRI (10.07.17): L4-5: disc extrusion, compression of both L5 nerve roots L5-S1: disc extrusion and compression of thecal sac and left S1 nerve root	2	3	Existing Korean medicine continued and nerve block performed	3 nerve blocks, 3 rounds of FIMS, and herbal medicine over 3 months but pain persisted. No further follow-up data
Outpatient	M	34	L-spine HIVD	Other specified intervertebral disc disorders	CMT, acupuncture, pharmacopuncture, cupping, herbal medicine	24	Increased lower back and lower limb radiating pain	L-spine MRI (09.06.25): disc protrusion in L4-5 and L3-4	L-spine MRI (11.02.15): disc protrusion in L4-5 and L3-4	2	3	Existing Korean medicine continued, nerve block and oral Western analgesia	One nerve block over 3 months combined with herbal medicine; pain mildly improved
Outpatient	M	62	Hypertension, Pneumothorax	Intervertebral disc disorder, unspecified	CMT, acupuncture, pharmacopuncture, cupping, herbal medicine	3	Increased lower back and lower limb radiating pain	L-spine MRI (10.06.07): disc extrusion in L1-2 and L2-3 Bulging disc in L3-4 and L4-5 and L5-S1	None	2	3	Existing Korean medicine treatment continued and OS injection added	10.06.19 OS raised suspicion for an L2-3 tumour, and advanced testing recommended. No further follow-up data.

Outpatient	M	37	-	Intervertebral disc disorder, unspecified	CMT, acupuncture, pharmacopuncture, cupping, herbal medicine	1	Increased lower back and lower limb radiating pain	L-spine MRI (10.07.09): disc protrusion in L3-4 disc extrusion in L4-5 and L5-S1	None	2	3	Existing Korean medicine treatment continued and nerve block performed	Herbal medicine added for 5 months, pain reduced
Outpatient	M	30	L-spine HIVD-nerve-block, Cholinergic urticaria	Intervertebral disc disorder, unspecified	CMT, acupuncture, pharmacopuncture, cupping, herbal medicine	16	Increased lower back and lower limb radiating pain	L-spine MRI (11.04.19): disc extrusion at L4-5	None	2	3	Existing Korean medicine continued and nerve block performed (excluding CMT)	Nerve block and herbal medicine continued for 1 year but pain persistent
Outpatient	F	55	L-spine HIVD-nerve-block, DM injection	Intervertebral disc disorder, unspecified	CMT, acupuncture, pharmacopuncture, cupping, herbal medicine	1	Increased lower back and lower limb radiating pain	L-spine MRI (10.12.20, another hospital): L2-3 and L3-4 and L4-5 extrusion with upward migration	None	2	3	Observation of progress	No follow-up data
Outpatient	M	69	L-spine stenosis, BPH	Intervertebral disc disorder, unspecified	CMT, acupuncture, pharmacopuncture, cupping, herbal medicine	1	Increased lower back and lower limb radiating pain	L-spine MRI (11.12.04): bulging disc at L2-3; disc extrusion with caudal migration in L4-5;	None.	2	3	Existing Korean medicine continued with oral Western analgesics	Persistent pain until 10 days later<br>No further follow-up data

								central spinal stenosis at L4-5; disc protrusion at L5-S1.					
Outpatient	F	44	L-spine HIVD	Lumbar and other intervertebral disc disorders with radiculopathy(G55.1*)	CMT, acupuncture, pharmacopuncture, cupping, herbal medicine	2	Increased lower back and lower limb radiating pain	L-spine MRI (12.01.20, another hospital): disc extrusion and central spinal stenosis in L4-5	None	2	3	Referred for nerve block	No follow-up data
Outpatient	F	28	-	Intervertebral disc disorder, unspecified	CMT, acupuncture, pharmacopuncture, cupping, herbal medicine	10	Increased lower back and lower limb radiating pain	L-spine MRI (11.09.21): disc protrusion at L4-5 disc protrusion and bulging disc at L5-S1	L-spine MRI (12.01.27): disc protrusion at L4-5; disc protrusion and bulging disc at L5-S1	2	3	Nerve block performed	Three nerve blocks over 3 months and surgery
Outpatient	M	58	Stenosis OP-laminectomy, BPH	Spinal stenosis, lumbar region	CMT, acupuncture, pharmacopuncture, cupping, herbal medicine	1	Increased right leg numbness	L-spine MRI (11.09.01): L4-5 spinal stenosis decompression postoperatively	None	1	3	Observation of progress	No follow-up data
Outpatient	M	37	-	Lumbar and other	CMT, acupuncture,	1	Exacerbated right leg	L-spine MRI (11.06.11, another hospital):	None	2	3	Observation of progress	Mildly improved 2 weeks later

				intervertebral disc disorders with radiculopathy(G55.1*)	pharmacopuncture, cupping, herbal medicine		numbness	disc protrusion at L4-5 and L5-S1					
Inpatient	M	52	L-spine HIVD	Lumbar and other intervertebral disc disorders with radiculopathy(G55.1*)	CMT, acupuncture, pharmacopuncture, cupping, herbal medicine	10	Increased lower back and lower limb radiating pain	L-spine MRI (14.09.16): L2-3 and L3-4 disc protrusion; L4-5 and L5-S1 disc protrusion with nerve root compression	L-spine MRI (19.09.04): disc protrusion at L2-3; disc bulging at L3-4 L4-5 disc protrusion with right L5 nerve root compression; disc bulge at L5-S1	2	3	Existing Korean medicine continued with herbal medicine (Kungak)	Improved 2 weeks later
Outpatient	F	42	-	Intervertebral disc disorder, unspecified	CMT, acupuncture, pharmacopuncture, herbal medicine	13	Exacerbated bilateral knee joint pain	None.	MRI of both knees '(12.10.18): R/O fat pad impingement syndrome; differential diagnosis: non-specific inflammatory change	2	3	Herbal medicine (Gwanjulhwan) and Western analgesics PO prescribed	Rheumatoid test performed 5 weeks later was negative; patient sought care at another hospital since

Outpatient	F	30	Wart	Cervical disc disorder, unspecified	CMT, acupuncture, pharmacopuncture, herbal medicine	1	Exacerbation of neck pain	CTL MRI (10.01.20) C4-5 and C5-6: suggestive of mild disc herniation	None	1	3	Current treatment continued and observation of progress	Improved 1 week later
Outpatient	F	49	-	Intervertebral disc disorder, unspecified	CMT, acupuncture, pharmacopuncture, herbal medicine	1	Increased low back pain	L-spine MRI (11.02.17): Disc bulging with annular tear at L4-5; disc protrusion at L5-S1	None	1	3	Current treatment continued and observation of progress	Pain improved 2 weeks later; no further follow-up data
Outpatient	M	62	BPH	Spondylolisthesis, lumbar region	CMT, acupuncture, pharmacopuncture, cupping, herbal medicine	6	Exacerbated lower back and left leg pain	L-spine MRI (16.12.17): T12-L1, L1-2, L2-3 and L-/4: bulging disc with thecal sac mild compression; L4-5: extrusion, foraminal stenosis, degenerative spondylolisthesis (grade 1)	None	1	3	Observation of progress	No follow-up data
Outpatient	F	30	-	Sprain and strain of lumbar spine	CMT, acupuncture, herbal medicine	1	Exacerbated low back pain	None	None	1	3	Current treatment continued and	Symptoms improved 3 days later

												observation of progress	
Outpatient	M	30	L-spine HIVD OP-discectomy	Other specified intervertebral disc disorders	CMT, acupuncture, pharmacopuncture, herbal medicine	38	Exacerbated left leg pain and transient sense of enervation	L-spine MRI (11.04.25): disc protrusion at L4-5 and L5-S1	None	2	2	Icepack, injection of analgesics, and current treatment continued	Mild improvement after bedrest immediately after treatment; improvement 3 days later
Inpatient	F	36	Orthostatic hypotension	Lumbar and other intervertebral disc disorders with radiculopathy(G55.1*)	CMT, acupuncture, pharmacopuncture, cupping, herbal medicine	15	Exacerbated low back pain	L-spine MRI (15.09.21): L3-4 protrusion; L4-5 disc extrusion and bulging; L5-S1 disc protrusion	None	1	3	Current treatment continued and observation of progress	Mild improvement after 6 weeks of Korean medicine

CMT: Chuna manipulation therapy, AE: adverse events, HIVD: herniated intervertebral disc, OP:operation, AVN: Avascular necrosis of the femoral head, TMJ: temporomandibular joint, R/O: reverse oblique, BPH: , MRI: magnetic resonance imaging

Table S4. Adverse events associated with rib fracture.

Visit type	Sex	Age, years	Comorbidity	Chief complaint	CMT session(s)	AE symptoms	Diagnostic test result	Severity	Causality	Post-AE treatment	Follow-up result
Outpatient	F	73	Hysterectomy, compression fracture	L-spine	1	Left flank pain	R/O incomplete fracture at left 3rd and 4th ribs on a radiograph	2	3	Observation of progress	No follow-up data
Outpatient	F	66	HIVD of L-spine OP, hypertension	L-spine, knee	4	Right flank pain	Right rib fracture on a radiograph	2	1	Herbal medicine (Jeopgol)	Symptoms improved 2 weeks later
Outpatient	F	70	-	L-spine	3	Left flank pain	Acute fracture at left 3rd and 4th ribs on a radiograph and MRI	2	1	Herbal medicine (Jeopgol)	Pain improved 4 weeks later
Outpatient	F	54	Hypertension, osteopenia	L-spine, C-spine	29	Left flank pain	R/O fracture, left 10th posterior arc on radiograph;	2	1	Herbal medicine (Goldagonghwan), oral and injected Western analgesics	Pain continued 1 week later, no further follow-up data
Inpatient	F	85	Arthritis, hypertension, left leg fracture, OP, osteopenia	L-spine, C-spine	6	Right flank pain	probable fracture of right 3rd rib on a radiograph	2	2	Observation of progress	Pain continued for 3 weeks later; no further follow-up data
Outpatient	F	72	HTN, DM	L-spine	2	Flank pain	WNL on a radiograph;<br>rib fracture on a bone scan	2	1	Observation of progress	Mild pain continued 2 weeks later;<br>no further follow-up data
Inpatient	F	68	-	L-spine	16	Right flank pain	Right 3rd rib fracture on a radiograph	2	1	Observation of progress	Mild pain continued 2 weeks

											later; no further follow-up data
Outpatient	F	78	Hyperlipidaemia	L-spine, Knee	3	Left flank pain	Left 5th and 6th rib fractures on a radiograph	2	1	Herbal medicine (Dangguisusan)	Mild pain continued 2 weeks later No further follow-up data
Outpatient	F	78	Hypertension, arrhythmia, urinary incontinence	L-spine	2	Right flank pain	Right 3rd rib fracture on a radiograph	2	1	Herbal medicine (Jeopgol)	Pain improved 2 weeks later
Outpatient	F	57	-	C-spine	20	Left flank pain	WNL on a radiograph; rib fracture on an ultrasonography scan	2	1	Physiotherapy, herbal medicine (Jeopgol)	Mild pain continued 4 months later; no further follow-up data
Inpatient	F	62	L-spine HIVD	L-spine	9	Right flank pain	Right 4th rib fracture on a radiograph	2	1	Observation of progress	Mild pain continued 4 weeks later

CMT: Chuna manipulation therapy, AE: adverse events, R/O: reverse oblique, HIVD: herniated intervertebral disc, OP: , WNL: within normal limits, HTN: hypertension, DM: diabetes mellitus

Table S5. Adverse events associated with falls.

Type of visit	Sex	Age, years	Comorbidity	Circumstances of fall	AE symptoms	Diagnostic test result	AE severity	Causality	Post-AE treatment	Follow-up result
Inpatient	F	62	Compression fracture at L5; left tympanum OP	Fell while changing to a supine position from a lateral position; fell on the right wrist	Right wrist pain	WNL on radiograph of the right wrist	1	1	Observation of progress	Pain resolved after 24 hours
Inpatient	M	73	Herniotomy, BPH C-spine HIVD OP, DM, Osteoporosis	Tension caused by trying to support the body when patient almost fell during CMT provoked pain	Existing symptoms exacerbated (right calf pain exacerbated)	No examination	1	1	Current intensive Korean medicine continued	Pain intensity reduced to below that before the onset of AE 5 days later
Outpatient	F	65	Hypertension	Fell while turning on the Chuna bed onto the left elbow, right hand, right rib	Left elbow pain (contusion)right rib pain	WNL on radiograph of the left elbow and ribs	1	1	Observation of progress	Symptoms improved 15 days later
Inpatient	F	54	-	Fell from the Chuna bed while changing posture during treatment; contusion of the left pelvis, left shoulder, and left knee	Left pelvic, left shoulder, left knee pain	WNL on both hips, left shoulder, and left knee on a radiograph	1	1	Observation of progress	No data after discharge
Outpatient	F	65	Right hip joint fracture; right Hip joint replacement OP	Fell while climbing up the Chuna bed for treatment and hit the right pelvis on the floor	None	No testing (patient was asymptomatic and refused radiography)	1	1	Observation of progress	Treatment continued via outpatient care for 3 months; no abnormalities observed

Outpatient	F	57	Hypertension	Fell due to sudden dizziness while sitting up after CMT[1] and sustained a contusion to the left side of the head	Dizziness, left forehead pain (Contusion)	V/S stable*; ENT and neurology visit recommended, no test	1	1	Sent home after 1 hour of bedrest Observation of progress	Contusion on the left forehead improved 5 days later.

CMT: Chuna manipulation therapy, AE: adverse events, R/O: reverse oblique, HIVD: herniated intervertebral disc, OP: operation, WNL: within normal limits

* V/S: 120/70-75-20-36.9, BST: 126 mg/dl, normally had dizziness when standing up from a sitting position.

Table S6. Adverse events associated with vertigo, chest pain, and discomfort.

AE symptoms	Type of visit	Sex	Age	Comorbidity	Chief complaint	CMT session(s)	Diagnostic test result	Severity	Causality	Post-AE treatment	Follow-up result
Headache, vertigo, nausea	Inpatient	F	38	-	LBP	6	Brain MRI + MRA findings normal	2	2	N/S 500 ml, herbal medicine, anti-inflammatory agents prescribed	Improved 5 days later
Chest pain	Outpatient	F	71	Hypertension, angina, hyperlipidaemia	Lumbar	1	Rib series radiograph and rib sonogram normal	1	1	Korean medicine treatment continued	Improved 1 month later
Chest pain	Inpatient	F	75	Hypertension	Lumbar	3	Rib series radiograph showed R/O compression fracture at T10–T12.	2	3	Korean medicine inpatient treatment continued	-
Discomfort	Outpatient	F	26	-	Cervical	1	-	1	1	-	-

CMT: Chuna manipulation therapy, AE: adverse events, MRI: magnetic resonance imaging, MRA: magnetic resonance angiography, N/S: Normal Saline
